# Development and Psychometric Evaluation of an Instrument Assessing Barriers to Growth Hormone Treatment (BAR-GHT)

**DOI:** 10.3389/fendo.2020.00084

**Published:** 2020-02-25

**Authors:** Martina de Zwaan, Josefine Fischer-Jacobs, Martin Wabitsch, Thomas Reinehr, Stefanie Meckes-Ferber, Ross D. Crosby

**Affiliations:** ^1^Department of Psychosomatic Medicine and Psychotherapy, Hannover Medical School, Hanover, Germany; ^2^Division of Pediatric Endocrinology and Diabetes, Department of Paediatrics and Adolescent Medicine, University Medical Center, Ulm, Germany; ^3^Vestische Kinder- und Jugendklinik Datteln, University Witten/Herdecke, Datteln, Germany; ^4^Clinical, Medical and Regulatory Department, Novo Nordisk Pharma GmbH, Mainz, Germany; ^5^Center for Biobehavioral Research, Sanford Health, Fargo, ND, United States

**Keywords:** growth hormone therapy, barriers, patient reported outcome measure, adherence, factor analysis

## Abstract

**Purpose:** This paper presents development and validation of a new patient reported outcome measure (PRO), the Barriers to Growth Hormone Therapy (BAR-GHT) in a patient (child/adolescent) and a parent version. The BAR-GHT was developed to measure problems and potential barriers to GHT.

**Methods:** The development and validation of the BAR-GHT was conducted according to the Food and Drug Administration (FDA) Guidance on the development of PROs. Concept elicitation included a literature review and open-ended interviews with young patients, parents, and clinical experts. Qualitative data were analyzed based on grounded theory principles and draft items were rated in terms of their importance and clarity. The instruments underwent psychometric validation in a German clinic-based patient population of children and adolescents who inject themselves and in a parent sample who inject their child. The statistical analysis plan included exploratory factor analysis, reliability, and validity.

**Results:** 29 patients, 22 parents, and 4 clinical experts participated in the concept elicitation, 156 children and adolescents aged 8–18 years and 146 parents completed the validation study. Exploratory factor analysis resulted in six domains: Fear, Public Embarrassment, Annoyance, Daily Routine, Supplies, and Travel. Internal consistencies and test-retest reliabilities of the total score of both the patient version and the parent version were >0.8. Convergent and discriminant validity was demonstrated.

**Conclusions:** The final 19-item BAR-GHT for patients aged 8–18 years and the 16-item version for parents can be considered reliable and valid PROs of barriers to GHT.

**Clinical Trial Registration:** www.ClinicalTrials.gov, identifier: NCT03672617.

Universal Trial Number (UTN) of the International Clinical Trials Registry Platform (ICTRP, www.who.int): U1111-1210-1036.

## Introduction

The main indications for treatment with human growth hormone (GH) in children and adolescents in Europe are growth hormone deficiency (GHD), Turner syndrome (TS), and small for gestational age (SGA). In Germany, about 3% of the children in the population are diagnosed with short stature (1). Children with short stature suffer from physical, social, and psychological impairments (2, 3). It is the aim of growth hormone therapy (GHT) to enable adequate growth during childhood and adolescence and to obtain an adult height within the normal range. GHT necessitates long-term treatment in order to achieve the desired outcome (4).

A major cause of suboptimal outcome of GHT is non-adherence (5–8). Growth velocity is significantly lower in children with poor adherence to GHT (5). Additionally, non-adherence may lead to poorer health outcomes (e.g., body composition), and reduced quality of life (9). It has been estimated that about a quarter of the patients who begin GHT discontinue it before completion of growth and thus many children do not attain their target adult height (9). Moreover, it has been shown that up to 82% of patients do not adhere to their daily therapy but miss out at least some GH doses. Adherence to regular daily injections of GH has been shown to decline with the duration of therapy. After 1 year of GHT only 67% of pediatric patients and 54% of adult patients fill their prescription. Potential factors associated with poor adherence to GHT have been summarized by Fisher and Acerini (6) and more recently by Graham et al. (7) as medication issues (e.g., side effects), scheduling issues (e.g., social convenience, away from home, traveling), family issues (e.g., responsibility for application, transition phase), and a variety of cognitive/emotional issues (e.g., lack of understanding, fear of needles, inadequate family support, denial). In a large Italian study with 1,007 children and adolescents aged between 6 and 16 years the most frequently reported reasons for missing a dose were being away from home, forgetfulness, not feeling well, and pain (10).

There is a need for effective interventions to improve adherence to GHT addressing the multiple reasons which may lead to non-adherence over time of treatment. In order to enable the clinicians to tailor such interventions, the individual patient's barriers to GHT or factors associated with non-adherence need to be known. Also, it is important to elicit the patients' own experiences (11). So far, a treatment-specific, patient-generated, and psychometrically sound measure that systematically captures treatment barriers to GHT is not available.

We developed an instrument according to guidance from the US Food and Drug Administration (FDA) on the use of patient-reported outcome (PRO) measures (12) assessing underlying barriers that may predispose the patients to low adherence. Two questionnaires were developed: A self-report for children and adolescents 8–18 years of age who administer GH themselves (self-injections) and an observer report for parents of infant and young patients aged 0–18 years who administer the GH to their child. Open-ended interviews including patients, parents and pediatric endocrinologists were the basis of concept elicitation. The instrument was developed in German for self-administration with a paper-based collection method.

## Materials and Methods

### Instrument Development

#### Concept Elicitation and Item Generation

Concept elicitation data was gathered from four sources: a review of the current literature regarding barriers to adherence with GHT, as well as open-ended interviews with young patients, parents and clinical experts. Data were collected iteratively so that completed interviews were used to guide and inform subsequent interviews.

The literature search was conducted using the PubMed (U.S. National Library of Medicine) and PsychInfo (American Psychological Association) databases. The following keywords were used: “GH,” “deficiency,” “treatment.” These terms were crossed with “adherence,” “compliance,” “persistence,” “discontinuation,” and “satisfaction.”

A convenience sample of patients and parents were recruited in four centers of pediatric endocrinology with recruitment allowing for variations in population characteristics such as age, sex, and disease (Table 1).

**Table 1:** Number of participants in open-ended interviews and cognitive debriefings.

		**Open-ended Interviews (N)**	**Cognitive debriefing (N)**	**Total**
**Patients**				
Children	Male <13 years	4	4	8
	Female <12 years	6	—	6
Adolescents	Male 13–18 years	6	1	7
	Female 12–18 years	5	3	8
**Parents**		18	4	22
**Clinical experts**		4	—	4

An interview guideline was developed based on literature review. The open-ended interviews targeted participants' personal experience with potential problems encountered with GHT that might be associated with non-adherence. Following the concept of data saturation, interviews were conducted until no new relevant and important information emerged. Saturation was achieved separately for each of the groups of participants. The patient, parent and expert interviews each lasted between 20 and 60 min.

The interviews were audiotaped, transcribed verbatim, and analyzed based on grounded theory principles of qualitative data analysis using MAXQDA 12^©^ software. Individual interview statements were extracted, summarized, and grouped into categories. This was done by one person (JF-J) and the logic of the categories was checked by a second researcher (MZ). Finally, items reflecting the categories were generated using the language of the participants and age-related vocabulary.

#### Cognitive Debriefing

The first version of the BAR-GHT which contained 59 items was cognitively debriefed by patients and parents meeting the same eligibility criteria as for the concept elicitation. Cognitive debriefing is a qualitative research tool used to determine whether concepts and items are understood by patients in the same way that instrument developers intend. Also, the importance and clarity of every item were rated on a 3-point Likert scale (0 = not important/difficult to understand, 1 = important/understandable, 2 = very important/easy to understand). Revisions to the measure were made according to the debriefing findings to create a validation ready version of the instrument. Items were dropped if they were reported as not relevant. The child/adolescent version contained 34 items and the parallel parent version contained 29 items. There were no comparable parent items for 3 questions on the child/adolescent version of the BAR-GHT. A recall period of 3 months was selected to cover the time between physician visits.

Six additional questions were developed to assess the occurrence and frequency of missed injections and about who is administering the injections.

#### Scoring

A five-point Likert scale was used as a response scale ranging from 1 (“not true/never”) to 5 (“always true”). The comprehension of these response scale categories was also assessed in the cognitive debriefing. Mean scores of each scale were calculated and transformed into values between 0 and 100 with lower values indicating the presence of more barriers.

### Validation Phase

#### Participants and Procedures

Two groups of participants were included: children and adolescents aged 8–18 years treated with GHT due to idiopathic GHD, multiple pituitary hormone deficiency (MPHD/organic GHD), SGA or TS who self-inject GH and parents who administer the injection. The patient group had to be on prescribed daily GHT for at least 6 months before the screening visit.

Signed informed consent was obtained by the parents and the child/adolescent before any study-related activities. The study was approved by the respective ethics committees of the participating centers.

Exclusion criteria were (1) learning difficulty or insufficient German language skills precluding adequate understanding or cooperation, (2) previous participation in this study, and (3) treatment of the child/adolescent with any investigational drug within 30 days prior to enrolment into the study.

Eighteen centers for pediatric endocrinology participated. Potential participants were identified by the pediatric endocrinologist at a routine clinic visit. Patients and parents completed the questionnaires during the clinic visit. For test-retest reliability assessment the BAR-GHT and a pre-stamped envelope were sent to all participants by mail 2 weeks later.

The instrument was developed in German for self-administration with a paper-based collection method. The German version was translated into English language with the help of a translation office. Back-translation was performed by an independent bilingual licensed translator in order to uncover potential ambiguity or misunderstanding in the first translation. The English version was not validated.

#### Validation Battery Measures (Collateral Measures)

##### Medication adherence report scale (MARS)

The MARS is a five-item self-report instrument focusing on non-adherent behavior (e.g., altering the dose of medication) on a 5-point scale (5 = “never,” 4 = “rarely,” 3 = “sometimes,” 2 = “often,” and 1 = “very often”) (13). Scores range from 5 to 25 with higher scores indicating higher adherence. The MARS was translated into German and validated in a sample of 523 patients with “chronic diseases and patients with risk factors of cardiovascular disease” (14). The MARS has been used in children and adolescents before [e.g., (15–17)]. For our study participants were asked to only consider GHT in their rating. Cronbach's α for the total score was 0.60 in the patient sample and 0.69 in the parent sample.

##### KIDSCREEN-27

The KIDSCREEN-27 (18) is a 27-item self-report measure of health-related quality of life in children and adolescents. The KIDSCREEN-27 provides five dimensional scores: Physical Well-Being, Psychological Well-Being, Autonomy & Parents, Peers & Social Support, and School Environment. The KIDSCREEN was completed by children and adolescents in the current study. Cronbach's α for the subscale scores ranged from 0.74 to 0.86.

##### Strengths and difficulties questionnaire (SDQ)

The SDQ is a self-report behavioral screening questionnaire for identifying behavioral or mental health problems in children and adolescents (19). Several age-adjusted versions of the SDQ are available for both children/adolescents (SDQ S11-17) and parents (SDQ P4-17). The SDQ provides a total score, as well as 5 subscale scores: Emotional Symptoms, Conduct Problems, Hyperactivity/Inattention, Peer Relationship Problems, and Prosocial Behavior. The SDQ was completed by both patients and parents. Cronbach's α for the total score and the subscale scores ranged from 0.54 (conduct problems) to 0.75 (hyperactivity) in the patient sample and 0.63 (conduct problems) to 0.82 (hyperactivity) in the parent sample.

Additionally, the following patient related parameters were collected at baseline: age and gender of the child/adolescent; reason, duration, and dose of GH-treatment; administrators of injections; education of child/adolescent; education of parents; family status (number of siblings, marital status). Parents filled out the SF-36 Health Survey, in order to assess their own functional health and well-being. Two summary measures were computed based on population norms: the physical component summary (PCS) and the mental component summary (MCS) score (20).

#### Statistical Analyses

BAR-GHT item analyses were conducted separately for child/adolescent and parent versions, including descriptive information such as mean, median, standard deviation, range, and skew. Subsequent analyses were based on available data. Given the very low rates of missing data in both the child/adolescent (0–3.8%) and parent (0–0.7%) version, we elected not to impute missing data. Spearman correlations were calculated between items to identify those items with high inter-correlations.

Exploratory factor analysis (EFA) was then conducted in the child/adolescent version using principal axis extraction and Promax oblique rotation. Items with low (<0.40) on-factor loadings or high (>0.30) cross-loadings were removed and analyses re-run in an iterative sequence. When a final factor structure was identified, sensitivity EFA was conducted using weighted least squares estimation with mean and variance adjustment (to account for ordinal variables) and Geomin oblique rotation. Parallel analysis was performed to confirm the number of factors. EFA analyses were repeated in the parent sample using only those items (where possible) that paralleled those from the final child/adolescent version. BAR-GHT scores were created for both child/adolescent and parent versions based upon a 0 (worst possible) to 100 (best possible) scoring, where at least 50% of the items within that scale had valid responses.

Cronbach's alpha coefficients were used to evaluate internal consistency of BAR-GHT scales and two-way mixed-effects intraclass correlations coefficients (ICC) were used to evaluate test-retest reliability. Reliability coefficients >0.60 were seen as sufficient. Spearman correlation coefficients were calculated between BAR-GHT scales and collateral measures to evaluate convergent/discriminant validity. Known-groups validity was evaluated by comparing adherence groups based on the MARS total score (25/24/<23) and by comparing those that report missing vs. not missing an injection in the last month. Analyses were conducted using SPSS version 25 and Mplus 8.1. Significance levels were set at 0.05 for all analyses.

## Results

### Study Participants

The child/adolescent sample consisted of 156 participants, including 101 (64.7%) boys and 55 (35.3%) girls. Mean age of participants was 12.7 years (*SD* = 2.1, range = 8.0–17.5), and the mean duration of GH treatment was 5.6 years (*SD* = 3.0, range = 0.5–12.7). Diagnoses included 96 participants (61.5%) with idiopathic GHD, 14 (9.0%) with MPHD, 33 (21.2%) with SGA, 8 (5.1%) with TS, and 5 (3.2%) not reporting a diagnosis. A total of 134 (85.9%) children/adolescents completed the test-retest assessment.

The parent sample consisted of 146 parents who administer GH to their child/adolescent. The target children of these parents included 90 (61.6%) boys and 56 (38.4%) girls with a mean age of 9.7 years (*SD* = 2.7, range = 4.0–16.5). The majority of parents were married (*N* = 104, 71.2%) or living with a partner (*N* = 13, 8.9%). The vast majority (>95%) of parents and their spouses/partners were working full or part time. A total of 126 (86.3%) of parents completed the test-retest assessment.

Table 2 presents descriptive information on key collateral measures in the child/adolescent and parent samples.

**Table 2:** Descriptive information on baseline assessments in the child/adolescent and parent samples.

**Assessment**	**Children/Adolescents** **(*N* = 156)**	**Parents** **(*N* = 146)**
MARS total score	23.7 (1.7)	24.3 (1.4)
SDQ emotional difficulties	2.1 (1.9)	2.6 (2.0)
SDQ conduct problems	1.7 (1.6)	2.2 (1.9)
SDQ hyperactivity	3.7 (2.4)	4.4 (2.8)
SDQ total	9.6 (5.4)	11.4 (6.8)
Kidscreen-27 psychological well-being	56.9 (11.4)	—
SF-36 physical component scale	—	52.5 (6.5)
SF-36 mental component scale	—	51.8 (8.0)
BAR-GHT scales^*^		
Fear	89.1 (16.5)	78.8 (23.7)
Travel	82.5 (23.8)	82.0 (22.1)
Supplies	96.3 (10.6)	97.6 (8.6)
Public embarrassment	74.3 (23.9)	78.0 (25.9)
Annoyance	72.5 (25.1)	67.1 (28.9)
Daily routine	89.4 (18.8)	91.4 (15.6)
Total	83.5 (12.2)	80.8 (14.3)

*Cell entries represent mean (SD)*.

* *lower scores indicate less compliant responses = more perceived barriers*.

*BAR-GHT, barriers to growth hormone treatment; MARS, medication adherence report scale; SDQ, strengths and difficulties questionnaire; SF-36, short form health survey*.

### Exploratory Factor Analysis

Table 3 presents factor loadings for the child/adolescent sample in normal text. Six factors were identified utilizing 19 items. All on-factor loadings were >0.40, while all cross-loadings were <0.30. Based upon item content, the following factors were identified. Factor 1, labeled as Fear, consists of four items relating to the fear and/or difficulty of self-injecting. Factor 2, labeled as Travel, consists of three items relating to the practical difficulties of injecting while traveling away from home. Factor 3, labeled as Supplies, consists of three items relating to running out of GH or injection supplies. Factor 4, labeled as Public Embarrassment, consists of four items relating to the difficulties and embarrassment of having others know that you inject growth hormone. Factor 5, labeled as Annoyance, consists of three items relating to the dislike or bother associated with injecting GH. Finally, Factor 6, labeled as Daily Routine, consists of two items relating to injections disrupting routine daily activities. A comparable six-factor structure was identified using an ordinal EFA model. Parallel analysis confirmed the six-factor structure.

**Table 3:** Exploratory factor analysis of BAR-GHT baseline items in the child/adolescent sample and parent samples.

**BAR-GHT question**	**Factor loadings**					
	**Factor 1**	**Factor 2**	**Factor 3**	**Factor 4**	**Factor 5**	**Factor 6**
I am afraid of injecting.	**0.94**	0.01	−0.01	−0.05	−0.07	−0.02
(*My child is afraid of the injections*)	**(*****0.98*****)**	(–*0.05*)	(*0.01*)	(*0.09*)	(–*0.05*)	(–*0.12*)
I am afraid that the injection will hurt.	**0.86**	0.06	0.03	0.09	−0.07	0.03
(*My child is afraid that the injection will hurt*)	**(*****0.71*****)**	(–*0.02*)	(*0.03*)	(*0.03*)	(*0.20*)	(–*0.14*)
It is hard for me to find the right spot for the injection.	**0.60**	−0.08	−0.02	0.03	0.04	0.12
	**—**	—	—	—	—	—
I can't bring myself to give myself the injection.	**0.53**	−0.00	0.02	−0.05	0.12	−0.05
*(My child cannot bring himself/herself to be injected)*	**(*****0.56*****)**	(*0.06*)	(*0.00*)	(−0.10)	(–*0.27*)	(*0.20*)
When I spend the night somewhere else, I may forget to give myself the injection.	−0.07	**0.94**	−0.05	−0.03	0.06	−0.04
*(When my child spends the night somewhere else (with friends, relatives), the injections may be forgotten)*	(–*0.04*)	**(*****0.53*****)**	(–*0.07*)	(–*0.03*)	(*0.08*)	(*0.02*)
When I spend the night somewhere else (with friends, relatives), I may not have the growth hormone with me.	0.01	**0.93**	0.02	0.07	−0.05	0.04
	—	**—**	—	—	—	—
When I'm on vacation, it may not have the growth hormone with me.	0.15	**0.42**	−0.01	−0.16	0.23	−0.06
*(When we are on vacation, we may not have the growth hormone with us)*	(–*0.12*)	**(*****0.52*****)**	(–*0.01*)	(–*0.04*)	(*0.08*)	(*0.11*)
I have run out of needles or the pen.	−0.06	0.13	**0.86**	0.01	−0.14	0.25
*(We have run out of needles or the pen)*	(*0.06*)	(*0.05*)	**(*****0.88*****)**	(*0.02*)	(–*0.03*)	(–*0.03*)
I have run out of growth hormone.	0.04	−0.05	**0.75**	0.13	0.09	−0.15
(*We have run out of growth hormone)*	(–*0.03*)	(*0.00*)	**(*****0.95*****)**	(–*0.03*)	(*0.03*)	(*0.01*)
I forgot to refill the pen.	0.03	−0.13	**0.60**	−0.13	0.30	−0.12
	—	—	**—**	—	—	—
I do not want to tell others why I give myself injections	0.06	0.06	0.09	**0.72**	−0.07	−0.18
*(My child does not want to tell others why he/she gets injections)*	(*0.18*)	(*0.20*)	(*0.01*)	**(*****0.76*****)**	(–*0.27*)	(*0.01*)
I feel ashamed to give myself the injection in front of someone else.	−0.03	0.02	−0.08	**0.66**	0.21	−0.16
*(My child feels ashamed to get injected in front of others)*	(–*0.12*)	(–*0.21*)	(*0.05*)	**(*****0.72*****)**	(*0.09*)	(*0.09*)
I do not want others see the bruises from the injections.	0.03	−0.10	0.02	**0.52**	0.07	0.02
*(My child does not want others to see the bruises from the injections)*	(*0.10*)	(*0.07*)	(–*0.06*)	**(*****0.64*****)**	(–*0.06*)	(–*0.05*)
I don't want to get so much attention from others because I give myself injections.	−0.08	−0.06	−0.00	**0.51**	−0.16	0.28
*(My child does not want to get so much attention from others for getting injections)*	(–*0.16*)	(–*0.11*)	(*0.01*)	**(*****0.67*****)**	(*0.23*)	(–*0.07*)
It bothers me that I have to inject the growth hormone every day.	−0.03	−0.01	0.07	0.05	**0.79**	0.20
*(It bothers/annoys my child that the growth hormone must be injected every day)*	(*0.15*)	(–*0.02*)	(*0.03*)	(–*0.04*)	**(*****0.76*****)**	(*0.14*)
I don't feel like injecting myself.	−0.01	0.07	0.14	−0.04	**0.73**	−0.11
(*My child does not want to get injected*).	(*0.29*)	(*0.05*)	(*0.02*)	(*0.03*)	**(*****0.70*****)**	(–*0.13*)
I don't like to have to inject myself every day.	0.04	0.07	−0.12	0.17	**0.62**	0.29
*(My child does not like to have to be injected every day)*	(*0.26*)	(*0.17*)	(−0.08)	(*0.04*)	**(*****0.70*****)**	(–*0.06*)
I feel limited by the injections.	0.10	−0.05	−0.02	−0.01	0.03	**0.76**
*(My child feels limited/restricted by the injections)*.	(–*0.01*)	(*0.02*)	(*0.00*)	(*0.14*)	(*0.15*)	**(*****0.57*****)**
The injections bother me in my daily activities.	−0.04	0.01	0.01	−0.13	0.10	**0.60**
*(My child feels that the injections disrupts his/her daily routine)*	(–*0.02*)	(*0.08*)	(–*0.02*)	(–*0.06*)	(–*0.03*)	**(*****0.82*****)**

*Normal text and factor loadings are for the child/adolescent sample. Italicized text and factor loadings in parentheses are for the parent sample. Bold factor loadings indicate item loadings on primary factor. — indicates no parallel parent question. BAR-GHT, barriers to growth hormone treatment*.

EFA loadings for the parent sample are presented in Table 3 in the italicized text in parentheses. One child/adolescent item (If I stay elsewhere overnight, it may be that I do not have the growth hormone with me) was not included on the parent form. Two additional items (It is difficult for me to find the right spot for injecting; I forgot to refill the pen) had poor factor loadings and were removed from the model. As in the child/adolescent model, six factors were identified consisting of a total 16 items. Also consistent with the child/adolescent model, all on-factor loadings were >0.40, while all cross-loadings were <0.30. Sensitivity analyses using an ordinal EFA model produced a comparable structure. Parallel analysis indicated a five-factor structure. Given the better interpretability of the six factor parent structure along with the comparable factor structure of the six-factor child/adolescent version, we elected to retain the six-factor parent structure for subsequent analyses.

Scale scores were calculated separately for each BAR-GHT scale assuming equal weighting of items using a 0–100 format, with 0 indicating the least compliant and 100 the most compliant response. Thus, lower scores indicate more perceived barriers. Scores were calculated when at least 50% of the items on the scale had a valid response. A total score was calculated using items from all scales. Descriptive information on BAR-GHT scale scores for the child/adolescent and parent versions is shown in Table 2.

### Missing Data

All items of both final versions (child/adolescents and parents) had very few missing data rates. Missing data on BAR-GHT items ranged from 0 to 3.8% in the child/adolescent sample and 0 to 0.7% in the parent sample. Most items showed a skew to the left, indicating a preposition for few barriers.

### Reliability

Reliability coefficients for BAR-GHT scales in the child/adolescent sample are presented in Table 4. Alpha coefficients were above 0.70 for all scales except Public Embarrassment (0.66) and Daily Routine (0.62). All test-retest coefficients were above 0.70 except Supplies (0.27) and Daily Routine (0.65). In the test-retest sample, significant improvements were noted from baseline to follow-up on Travel (*p* = 0.04), Supplies (*p* = 0.04), Public Embarrassment (*p* = 0.02), Annoyance (*p* = 0.03), and Total (*p* < 0.01) based upon Wilcoxon non-parametric tests. These significant improvements may have resulted in diminished test-retest coefficients for these scales based upon true changes. With the exception of the test-retest reliability for the Supplies scale, the reliability of the BAR-GHT scales for the child/adolescent sample appears to be adequate.

**Table 4:** Internal consistency and test-retest reliability of the BAR-GHT scales.

**BAR-GHT scale**	**Reliability**	**Sample**	
		**Child/adolescent**	**Parent**
Fear	Internal consistency (Cronbach's alpha)	0.81	0.82
	Test-retest (ICC)	0.74	0.80
Travel	Internal consistency (Cronbach's alpha)	0.82	0.49
	Test-retest (ICC)	0.92	0.84
Supplies	Internal consistency (Cronbach's alpha)	0.77	0.91
	Test-retest (ICC)	0.27	0.77
Public embarrassment	Internal consistency (Cronbach's alpha)	0.66	0.77
	Test-retest (ICC)	0.78	0.80
Annoyance	Internal consistency (Cronbach's alpha)	0.84	0.90
	Test-retest (ICC)	0.76	0.81
Daily routine	Internal consistency (Cronbach's alpha)	0.62	0.72
	Test-retest (ICC)	0.65	0.59
Total	Internal consistency (Cronbach's alpha)	0.81	0.82
	Test-retest (ICC)	0.82	0.84

*ICC, Intraclass correlations coefficient*.

Reliability coefficients for BAR-GHT scales in the parent sample are also presented in Table 4. Alpha coefficients were above 0.70 for all scales except for Travel (0.49). All test-retest coefficients were above 0.70 except for Daily Routine (0.59). Based upon these findings, the reliability of the BAR-GHT scales for the parent sample appears to be adequate.

### Convergent/Discriminant Validity

Spearman correlations between BAR-GHT scales and collateral measures for the child/adolescent and parent samples are presented in Table 5. In the child/adolescent sample, all BAR-GHT scales correlated significantly with the MARS total score, with the highest correlations for Travel (rho = 0.65), and Total (rho = 0.51). BAR-GHT scale also correlated consistently with SDQ Emotional Difficulties and Kidscreen-27 Psychological Well-Being. In the parent sample, only the BAR-GHT Travel scale correlated significantly with the MARS Total score. However, several BAR-GHT scales, including the Total Score, correlated significantly with parent-rated SDQ Emotional Difficulties, SDQ Total score, and the SF-36 Mental Component scale score.

**Table 5:** Spearman correlations between BAR-GHT scales and collateral measures.

**Collateral measure**	**BAR-GHT scale**						
	**Fear**	**Travel**	**Supplies**	**Public embarrassment**	**Annoyance**	**Daily routine**	**Total**
MARS total	0.25^**^	0.65^**^	0.32^**^	0.24^**^	0.44^**^	0.17^*^	0.51^**^
	(*0.02*)	(0.46^**^)	(*0.15*)	(–*0.03*)	(*0.14*)	(–*0.03*)	(*0.12*)
SDQ emotional difficulties	−0.21^**^	−0.08	−0.25^**^	−0.26^**^	−0.26^**^	−0.26^**^	−0.35^**^
	(–0.28^**^)	(–*0.08*)	(–*0.10*)	(–0.27^**^)	(–0.17^*^)	(–0.31^**^)	(–0.30^**^)
SDQ conduct problems	−0.10	−0.15	−0.17^*^	−0.08	−0.14	−0.07	−0.18^*^
	(–*0.12*)	(–*0.09*)	(–*0.16*)	(–*0.10*)	(–*0.16*)	(–0.17^*^)	(–0.19^*^)
SDQ hyperactivity	−0.06	−0.15	−0.17^*^	−0.03	−0.03	−0.00	−0.07
	(–0.22^**^)	(–*0.11*)	(–*0.07*)	(–*0.13*)	(–*0.16*)	(–*0.10*)	(–0.25^**^)
SDQ total	−0.12	−0.19^*^	−0.21^**^	−0.15	−0.18^*^	−0.12	−0.27^**^
	(–0.24^**^)	(–*0.09*)	(–*0.14*)	(–0.21^*^)	(–0.17^*^)	(–0.21^*^)	(–0.29^**^)
Kidscreen-27 psychological well-being	0.26^**^	0.19^*^	0.17^*^	0.22^**^	0.32^**^	0.21^**^	0.36^**^
	(—)	(—)	(—)	(—)	(—)	(—)	(—)
SF-36 physical component	—	—	—	—	—	—	—
	(*0.02*)	(*0.09*)	(–*0.15*)	(*0.10*)	(–*0.07*)	(*0.09*)	(*0.01*)
SF-36 mental component	—	—	—	—	—	—	—
	(0.22^**^)	(*0.16*)	(0.21^*^)	(0.24^**^)	(*0.12*)	(*0.11*)	(0.29^**^)

* p < 0.05

** *p < 0.01*.

*Normal text correlations are for the child/adolescent sample. Italicized correlations in parentheses are for the parent sample*.

*— indicates measure not given in child/adolescent sample. (—) indicates measure not given in parent sample*.

*BAR-GHT, barriers to growth hormone treatment; MARS, medication adherence report scale; SDQ, strengths and difficulties questionnaire; SF-36, short form health survey*.

No significant associations were detected between the BAR-GHT scales and age, sex, and duration of GHT.

### Known-Groups Validity

Kruskal-Wallis non-parametric U-tests were used to compare adherence groups (MARS total = 25; *N* = 60 vs. MARS total = 24; *N* = 50 vs. MARS total <23; *N* = 42) participants on BAR-GHT scale scores in the child/adolescent and parent samples. In the child/adolescent sample, BAR-GHT scale scores were significantly (all *p*'s <0.02) different on all scales except Daily Routine, with higher BAR-GHT scores for the highest adherence group. In the parent sample, the only significant difference between adherence group was on Travel (*p* < 0.001), where higher BAR-GHT scores were found in the highest adherence group.

Mann-Whitney non-parametric U-tests were used to compare participants that reported missing vs. not missing an injection in the last month in both the child/adolescent and parent samples. In the child/adolescent sample, significant differences were found between those missing (*N* = 57) and not missing (*N* = 95) an injection in the last month on Travel (*p* < 0.001), Annoyance (*p* < 0.001), and Total (*p* < 0.01), with higher BAR-GHT scale scores for those not missing a dose. In the parent sample, significant differences were found between those missing (*N* = 37) and not missing (*N* = 108) an injection in the last month on Fear (*p* = 0.04), Travel (*p* < 0.001), Supplies (*p* = 0.02), Annoyance (*p* < 0.01), and Total (*p* < 0.01), with higher BAR-GHT scale scores for those not missing a dose.

## Discussion

The present study aimed to develop and evaluate a new treatment-specific instrument to assess potential barriers for adherence to GHT. The patient-reported outcome (PRO) measure was developed according to FDA guidelines (12). Two versions were created: one for children and adolescents aged 8–18 years who self-inject GH (self-report) and a parallel version for parents of children who are not administering the injections themselves (observer-report).

The finding of the factor analysis of the child/adolescent version revealed a 6-factor solution with 19 items loading on the dimensions Fear (4 items), Travel (3 items), Supplies (3 items), Public Embarrassment (3 items), Annoyance (3 items), and Daily Routine (2 items). The 6-factor structure of the 19-item children/adolescent version was confirmed in the parent version with 16 items (Fear 3 items, Travel 2 items, Supplies 2 items, Public Embarrassment 3 items, Annoyance 3 items, and Daily Routine 2 items).

Internal consistency of the total score was good in both versions (α >0.8) and acceptable to good for most of the subscale scores in both versions except for the Travel subscale of the parent version (α = 0.49). Overall, item numbers per subscale were low, which usually decreases Cronbach's alpha.

Test-retest reliability was good for the total score in both versions (α >0.8) and acceptable to good for most subscale scores except for the Supplies subscale scores in the child/adolescent version (0.2). Interestingly, in the test-retest sample, significant improvements were noted from baseline to follow-up on Travel (*p* = 0.04), Supplies (*p* = 0.04), Public Embarrassment (*p* = 0.02), Annoyance (*p* = 0.03), and Total (*p* < 0.01). These significant improvements may have resulted in diminished test-retest coefficients for these scales based upon true changes.

As expected, perceived barriers to GHT were associated with measures of adherence. Even though the BAR-GHT was not developed to measure adherence directly, there was a significant association with a large effect size (*r* = 0.51) between the MARS score and all of the BAR-GHT subscale scores and the total score for the child/adolescent version. In both, the child/adolescent and the parent version known-group validity (missing vs. not missing an injection in the last month) revealed significant differences in most of the subscales. Similar results were reported by Mohseni et al. (21) who reported that 16 probable barriers to GHT were significantly associated with another self-report adherence scale, the Morisky Medication Adherence Scale.

In the child/adolescent version we found significant correlations with psychological well-being (KIDSCREEN) and the emotional difficulties and conduct problems subscales of the SDQ indicating convergent validity. Overall, these associations were less strong in the parent version; however, the association between the SDQ total score and the BAR-GHT total score was comparable for both versions. Having higher levels of emotional difficulties seems to be associated with more perceived barriers regarding GHT. The perception of barriers to GHT and adherence to GHT are most likely multi-factorial phenomena. Especially in long-term illnesses preparing and organizing regular medicine use can be challenging and can be affected by mental problems. On the other hand chronic illness and the necessity of regular injections might also cause reduced quality of life and a decline in psychological well-being. (22).

Interestingly, we found no age differences between the BAR-GHT scales; thus the pattern of barriers did not differ between children and adolescents as suggested by Mohseni et al. (21).

In their systematic review Graham et al. (7) categorized potentially modifiable factors associated with treatment non-adherence amongst young patients receiving GHT according to the COM-B framework. The COM-B posits that human behavior (B) results from the interaction between psychological and physical capabilities (C), opportunities provided by the social and physical environment (O) and reflective and automatic motivation (M) (23). The COM-B framework has also been applied to categorize the mechanisms responsible for non-adherence to medication and can be used to facilitate the development of targeted interventions (24). Even though the BAR-GHT was not developed based on this framework, the sub-components of the COM-B are covered by subscales or individual questions within subscales. The subscales “Travel” and “Daily Routine” can be classified as automatic motivation factors as they describe lifestyle disruptions (7); however, “Travel” might also be classified as physical opportunity factor since it describes circumstances lying outside the individual which might hinder adherence. The “Annoyance” subscale is best classified as a reflective motivation factor. “Embarrassment” is a social opportunity factor as it describes fear of stigma and disclosure but might also have an effect on adherence by impacting confidence (reflective motivation). Two questions of the “Fear” subscale can be classified as physical opportunity factors (discomfort and pain associated with daily injection) and two as reflective motivation factors (low confidence regarding self-administration). The “Supplies” subscale can be classified as a psychological capability factor since it covers mainly failure to renew the prescription (7) and not logistical problems (such as cost, access, packaging) (24) which would fall into the physical opportunity sub-component. Thus, the BAR-GHT items could be classified within the COM-B framework, even though some subscales may not map directly onto a single sub-component.

## Limitations

When using child reported data in longitudinal studies, the cognitive development process of the children need to be considered (25). Opinions, feelings, and attitudes might change quickly resulting in score changes. In the current study, the child/adolescent sample showed significant improvements on five BAR-GHT scales across the 2 week test-retest interval, raising questions about test-retest reliability on some scales. Thus, in longitudinal quality of life research the use of parent-reported outcome has been recommended (26). The cross-sectional design limits inferences of causality. We cannot differentiate whether barriers to GHT influence adherence behavior or whether non-adherence causes patients to adjust their responses regarding barriers to justify their behavior. Another limitation is the use of self-report measures which are subject to patient recall bias and might be biased due to socially desirable response behavior.

In conclusion, the scales showed satisfactory reliability and adequate validity offering the opportunity to adequately assess potential barriers for adherence with GHT from the young patients' and the parents' perspective. Patients' experience of long-term medicines use varies, and instruments which measure barriers of medication use are seen as an essential component to improve care.

The study suggests that the BAR-GHT can be used in descriptive adherence studies that identify patient needs for care and can also be used in clinical trials aiming to improve adherence. It also allows for comparison between studies. Early identification of probable barriers to the adherence to GHT can guide health care providers to design proper treatment strategies. However, the ability of the BAR-GHT to detect change needs to be investigated.

## Data Availability Statement

The datasets for this study are available upon reasonable request from the corresponding author.

## Ethics Statement

The studies involving human participants were reviewed and approved by Hannover Medical School. The study was also approved by the respective Ethics Committees of all participating centers. Written informed consent to participate in this study was provided by the participants or the participants' legal guardian/next of kin.

## Author Contributions

MZ, SM-F, and RC designed the study. JF-J conducted the qualitative part of the study (instrument development). MW and TR were involved in data collection in both the qualitative (instrument development) and quantitative (validation) parts of the study. RC was responsible for the statistical plan and the analyses. MZ wrote the first draft of the manuscript. All authors contributed substantially.

### Conflict of Interest

The authors declare that this study received funding from Novo Nordisk. The funder had the following involvement with the study: Logistical support for data collection. RC is a consultant for Health Outcomes Solutions, Winter Park, Florida and an employee of Sanford Health.

## References

[1] Hoepffner W Pfaffle R Gausche R Meigen C Keller E. Early detection of growth disorders with the CrescNet system at the Leipzig treatment center. Dtsch Arztebl Int. (2011) 108:123–8. 10.3238/arztebl.2011.0123 21403802 PMC3055254

[2] Geisler A Lass N Reinsch N Uysal Y Singer V Ravens-Sieberer U. Quality of life in children and adolescents with growth hormone deficiency: association with growth hormone treatment. Horm Res Paediatr. (2012) 78:94–9. 10.1159/000341151 22907471

[3] Sandberg DE Gardner M. Short stature: is it a psychosocial problem and does changing height matter? Pediatr Clin North Am. (2015) 62:963–82. 10.1016/j.pcl.2015.04.009 26210627

[4] Ranke MB Wit JM. Reflections on the US guidelines on growth hormone and insulin-like growth factor-I treatment in children and adolescents. Horm Res Paediatr. (2016) 86:398–402. 10.1159/000452446 27898423

[5] Cutfield WS Derraik JG Gunn AJ Reid K Delany T Robinson E. Non-compliance with growth hormone treatment in children is common and impairs linear growth. PLoS ONE. (2011) 6:e16223. 10.1371/journal.pone.0016223 21305004 PMC3031542

[6] Fisher BG Acerini CL. Understanding the growth hormone therapy adherence paradigm: a systematic review. Horm Res Paediatr. (2013) 79:189–96. 10.1159/000350251 23635797

[7] Graham S Weinman J Auyeung V. Identifying potentially modifiable factors associated with treatment non-adherence in paediatric growth hormone deficiency: a systematic review. Horm Res Paediatr. (2018) 90:221–7. 10.1159/000493211 30522126

[8] Lass N Reinehr T. Low treatment adherence in pubertal children treated with thyroxin or growth hormone. Horm Res Paediatr. (2015) 84:240–7. 10.1159/000437305 26279278

[9] Rosenfeld RG Bakker B. Compliance and persistence in pediatric and adult patients receiving growth hormone therapy. Endocr Pract. (2008) 14:143–54. 10.4158/EP.14.2.143 18308651

[10] Bagnasco F Di Iorgi N Roveda A Gallizia A Haupt R Maghnie M. Prevalence and correlates of adherence in children and adolescents treated with growth hormone: a multicenter Italian study. Endocr Pract. (2017) 23:929–41. 10.4158/EP171786.OR 28614005

[11] Katusiime B Corlett S Reeve J Krska J. Measuring medicine-related experiences from the patient perspective: a systematic review. Patient Relat Outcome Meas. (2016) 7:157–71 10.2147/PROM.S102198 27785116 PMC5063133

[12] US Food and Drug Administration Guidance for Industry Patient-Reported Outcome Measures: Use in Medical Product Development to Support Labeling Claims. Rockville, MD (2009). 10.1186/1477-7525-4-79 PMC1629006 17034633

[13] Horne R Weinman J. Patients' beliefs about prescribed medicines and their role in adherence to treatment in chronic physical illness. J Psychosom Res. (1999) 47:555–67. 10.1016/S0022-3999(99)00057-4 10661603

[14] Mahler C Hermann K Horne R Ludt S Haefeli WE Szecsenyi J. Assessing reported adherence to pharmacological treatment recommendations. translation and evaluation of the medication adherence report scale (MARS) in Germany. J Eval Clin Pract. (2010) 16:574–9. 10.1111/j.1365-2753.2009.01169.x 20210821

[15] Garcia-Marcos PW Brand PL Kaptein AA Klok T. Is the MARS questionnaire a reliable measure of medication adherence in childhood asthma? J Asthma. (2016) 53:1085–9. 10.1080/02770903.2016.1180699 27177241

[16] Wehmeier PM Dittmann RW Banaschewski T. Treatment compliance or medication adherence in children and adolescents on ADHD medication inclinical practice: results from the COMPLY observational study. Atten Defic Hyperact Disord. (2015) 7:165–74. 10.1007/s12402-014-0156-8 25416667

[17] Alsous M Abu Farha R Alefishat E Al Omar S Momani D Gharabli A. Adherence to 6-mercaptopurine in children and adolescents with acute lymphoblastic leukemia. PLoS ONE. (2017) 12:e0183119. 10.1371/journal.pone.0183119 28877179 PMC5587295

[18] Ravens-Sieberer U Gosch A Abel T Auquier P Bellach BM Dür W. Quality of life in children and adolescents: a European public health perspective. Soz Praventivmed. (2001) 46:294–302. 10.1007/BF01321080 11759336

[19] Goodman R. The strengths and difficulties questionnaire: a research note. J Child Psychol Psychiatry. (1997) 38:581–6. 10.1111/j.1469-7610.1997.tb01545.x 9255702

[20] Ware JE Jr Kosinski M Bayliss MS McHorney CA Rogers WH Raczek A. Comparison of methods for the scoring and statistical analysis of SF-36 health profile and summary measures: summary of results from the medical outcome study. Med Care. (1995) 33(Suppl. 4):AS264–79. 7723455

[21] Mohseni S Heydari Z Qorbani M Radfar M. Adherence to growth hormone therapy in children and its potential barriers. J Pediatr Endocrinol Metab. (2018) 31:13–20. 10.1515/jpem-2017-0157 29216008

[22] Quitmann J Bloemeke J Silva N Bullinger M Witt S Akkurt I. Quality of life of short-statured children born small for gestational age or idiopathic growth hormone deficiency within 1 year of growth hormone treatment. Front Pediatr. (2019) 7:164. 10.3389/fped.2019.00164 31111024 PMC6501464

[23] Michie S van Stralen MM West R. The behaviour change wheel: a new method for characterising and designing behaviour change interventions. Implement Sci. (2011) 6:42. 10.1186/1748-5908-6-42 21513547 PMC3096582

[24] Jackson C Eliasson L Barber N Weinman J Applying COM-B to medication adherence. Eur Health Psychol. (2014) 16:7–17.

[25] Bloemeke J Silva N Bullinger M Witt S Dörr HG Quitmann J. Psychometric properties of the quality of life in short statured youth (QoLISSY) questionnaire within the course of growth hormone treatment. Health Qual Life Outcomes. (2019) 17:49. 10.1186/s12955-019-1118-9 30885197 PMC6423839

[26] le Coq EM Boeke AJ Bezemer PD Colland VT van Eijk JT. Which source should we use to measure quality of life in children with asthma: the children themselves or their parents? Qual Life Res. (2000) 9:625–36. 10.1023/A:1008977200176 11236853

